# Supplementary material for: In Silico Screening and Optimization of Cell-Penetrating Peptides Using Deep Learning Methods
Source: Biomolecules. 2023 Mar 13;13(3):522. doi: 10.3390/biom13030522 (PMC10046020; doi:10.3390/biom13030522)
Supplement: Supplementary file 1 [file biomolecules-13-00522-s001.zip › biomolecules-2162660_Supplementary information.pdf]

Supplementary information

Model 1

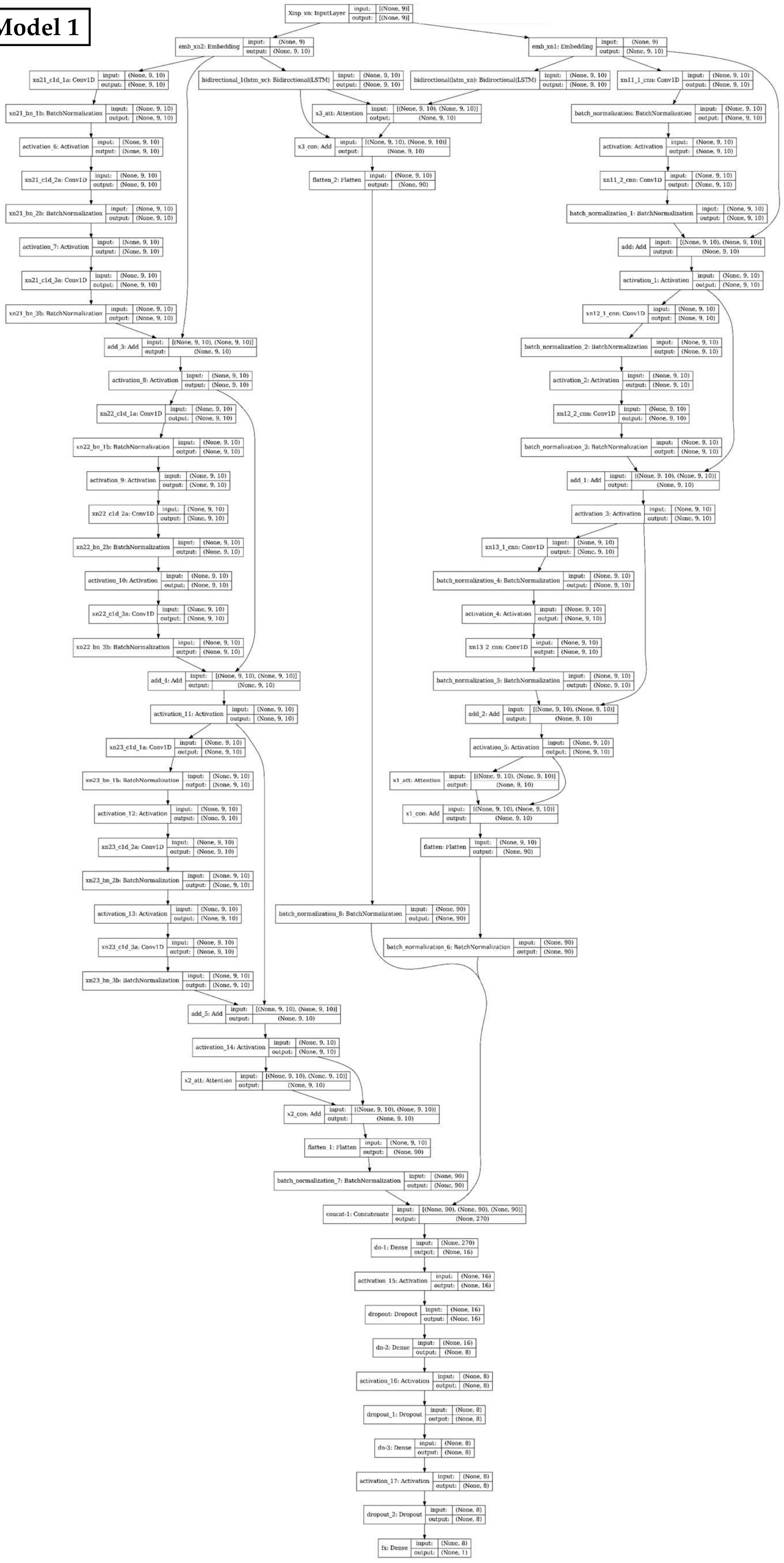

Model 2

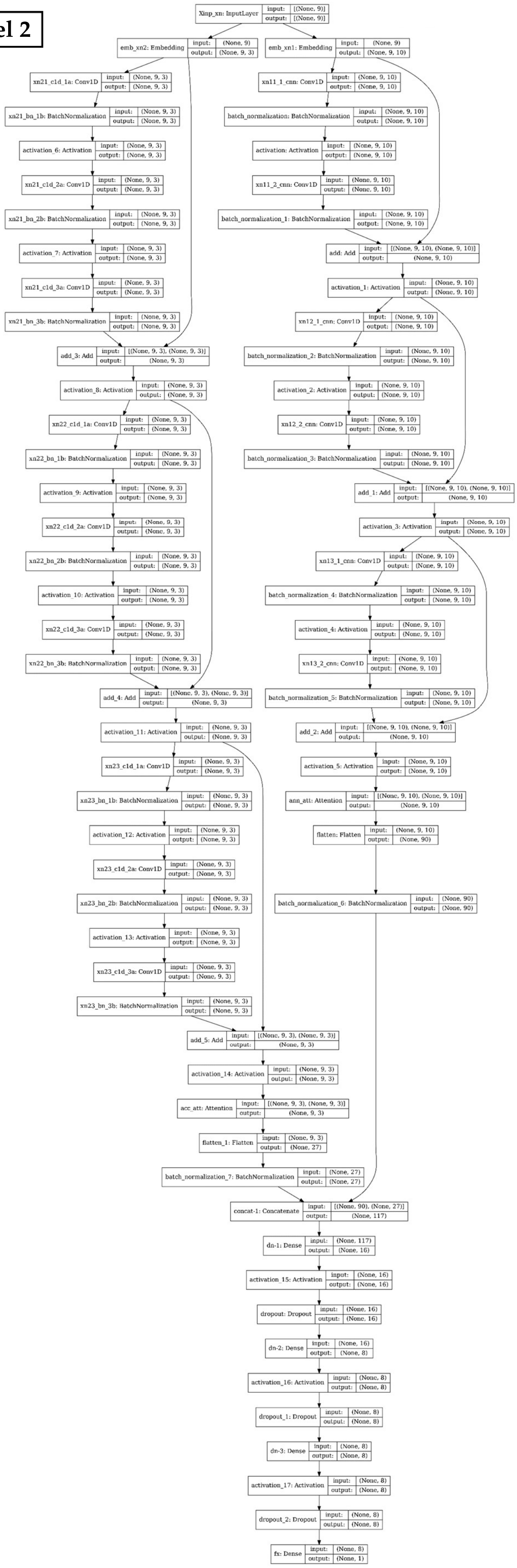
Xinp_xn: InputLayer input: [(None, 9)] output: [(None, 9)]
emb_xn2: Embedding input: (None, 9) output: (None, 9, 3)
emb_xn1: Embedding input: (None, 9) output: (None, 9, 10)
xn21_c1d_1a: Conv1D input: (None, 9, 3) output: (None, 9, 3)
xn21_bn_1b: BatchNormalization input: (None, 9, 3) output: (None, 9, 3)
activation_6: Activation input: (None, 9, 3) output: (None, 9, 3)
xn21_c1d_2a: Conv1D input: (None, 9, 3) output: (None, 9, 3)
xn21_bn_2b: BatchNormalization input: (None, 9, 3) output: (None, 9, 3)
activation_7: Activation input: (None, 9, 3) output: (None, 9, 3)
xn21_c1d_3a: Conv1D input: (None, 9, 3) output: (None, 9, 3)
xn21_bn_3b: BatchNormalization input: (None, 9, 3) output: (None, 9, 3)
add_3: Add input: [(None, 9, 3), (None, 9, 3)] output: (None, 9, 3)
activation_8: Activation input: (None, 9, 3) output: (None, 9, 3)
xn22_c1d_1a: Conv1D input: (None, 9, 3) output: (None, 9, 3)
xn22_bn_1b: BatchNormalization input: (None, 9, 3) output: (None, 9, 3)
activation_9: Activation input: (None, 9, 3) output: (None, 9, 3)
xn22_c1d_2a: Conv1D input: (None, 9, 3) output: (None, 9, 3)
xn22_bn_2b: BatchNormalization input: (None, 9, 3) output: (None, 9, 3)
activation_10: Activation input: (None, 9, 3) output: (None, 9, 3)
xn22_c1d_3a: Conv1D input: (None, 9, 3) output: (None, 9, 3)
xn22_bn_3b: BatchNormalization input: (None, 9, 3) output: (None, 9, 3)
add_4: Add input: [(None, 9, 3), (None, 9, 3)] output: (None, 9, 3)
activation_11: Activation input: (None, 9, 3) output: (None, 9, 3)
xn23_c1d_1a: Conv1D input: (None, 9, 3) output: (None, 9, 3)
xn23_bn_1b: BatchNormalization input: (None, 9, 3) output: (None, 9, 3)
activation_12: Activation input: (None, 9, 3) output: (None, 9, 3)
xn23_c1d_2a: Conv1D input: (None, 9, 3) output: (None, 9, 3)
xn23_bn_2b: BatchNormalization input: (None, 9, 3) output: (None, 9, 3)
activation_13: Activation input: (None, 9, 3) output: (None, 9, 3)
xn23_c1d_3a: Conv1D input: (None, 9, 3) output: (None, 9, 3)
xn23_bn_3b: BatchNormalization input: (None, 9, 3) output: (None, 9, 3)
add_5: Add input: [(None, 9, 3), (None, 9, 3)] output: (None, 9, 3)
activation_14: Activation input: (None, 9, 3) output: (None, 9, 3)
acc_att: Attention input: [(None, 9, 3), (None, 9, 3)] output: (None, 9, 3)
flatten_1: Flatten input: (None, 9, 3) output: (None, 27)
batch_normalization_7: BatchNormalization input: (None, 27) output: (None, 27)
xn11_1_cnn: Conv1D input: (None, 9, 10) output: (None, 9, 10)
batch_normalization: BatchNormalization input: (None, 9, 10) output: (None, 9, 10)
activation: Activation input: (None, 9, 10) output: (None, 9, 10)
xn11_2_cnn: Conv1D input: (None, 9, 10) output: (None, 9, 10)
batch_normalization_1: BatchNormalization input: (None, 9, 10) output: (None, 9, 10)
add: Add input: [(None, 9, 10), (None, 9, 10)] output: (None, 9, 10)
activation_1: Activation input: (None, 9, 10) output: (None, 9, 10)
xn12_1_cnn: Conv1D input: (None, 9, 10) output: (None, 9, 10)
batch_normalization_2: BatchNormalization input: (None, 9, 10) output: (None, 9, 10)
activation_2: Activation input: (None, 9, 10) output: (None, 9, 10)
xn12_2_cnn: Conv1D input: (None, 9, 10) output: (None, 9, 10)
batch_normalization_3: BatchNormalization input: (None, 9, 10) output: (None, 9, 10)
add_1: Add input: [(None, 9, 10), (None, 9, 10)] output: (None, 9, 10)
activation_3: Activation input: (None, 9, 10) output: (None, 9, 10)
xn13_1_cnn: Conv1D input: (None, 9, 10) output: (None, 9, 10)
batch_normalization_4: BatchNormalization input: (None, 9, 10) output: (None, 9, 10)
activation_4: Activation input: (None, 9, 10) output: (None, 9, 10)
xn13_2_cnn: Conv1D input: (None, 9, 10) output: (None, 9, 10)
batch_normalization_5: BatchNormalization input: (None, 9, 10) output: (None, 9, 10)
add_2: Add input: [(None, 9, 10), (None, 9, 10)] output: (None, 9, 10)
activation_5: Activation input: (None, 9, 10) output: (None, 9, 10)
ann_att: Attention input: [(None, 9, 10), (None, 9, 10)] output: (None, 9, 10)
flatten: Flatten input: (None, 9, 10) output: (None, 90)
batch_normalization_6: BatchNormalization input: (None, 90) output: (None, 90)
concat-1: Concatenate input: [(None, 90), (None, 27)] output: (None, 117)
dn-1: Dense input: (None, 117) output: (None, 16)
activation_15: Activation input: (None, 16) output: (None, 16)
dropout: Dropout input: (None, 16) output: (None, 16)
dn-2: Dense input: (None, 16) output: (None, 8)
activation_16: Activation input: (None, 8) output: (None, 8)
dropout_1: Dropout input: (None, 8) output: (None, 8)
dn-3: Dense input: (None, 8) output: (None, 8)
activation_17: Activation input: (None, 8) output: (None, 8)
dropout_2: Dropout input: (None, 8) output: (None, 8)
fx: Dense input: (None, 8) output: (None, 1)

Model 3

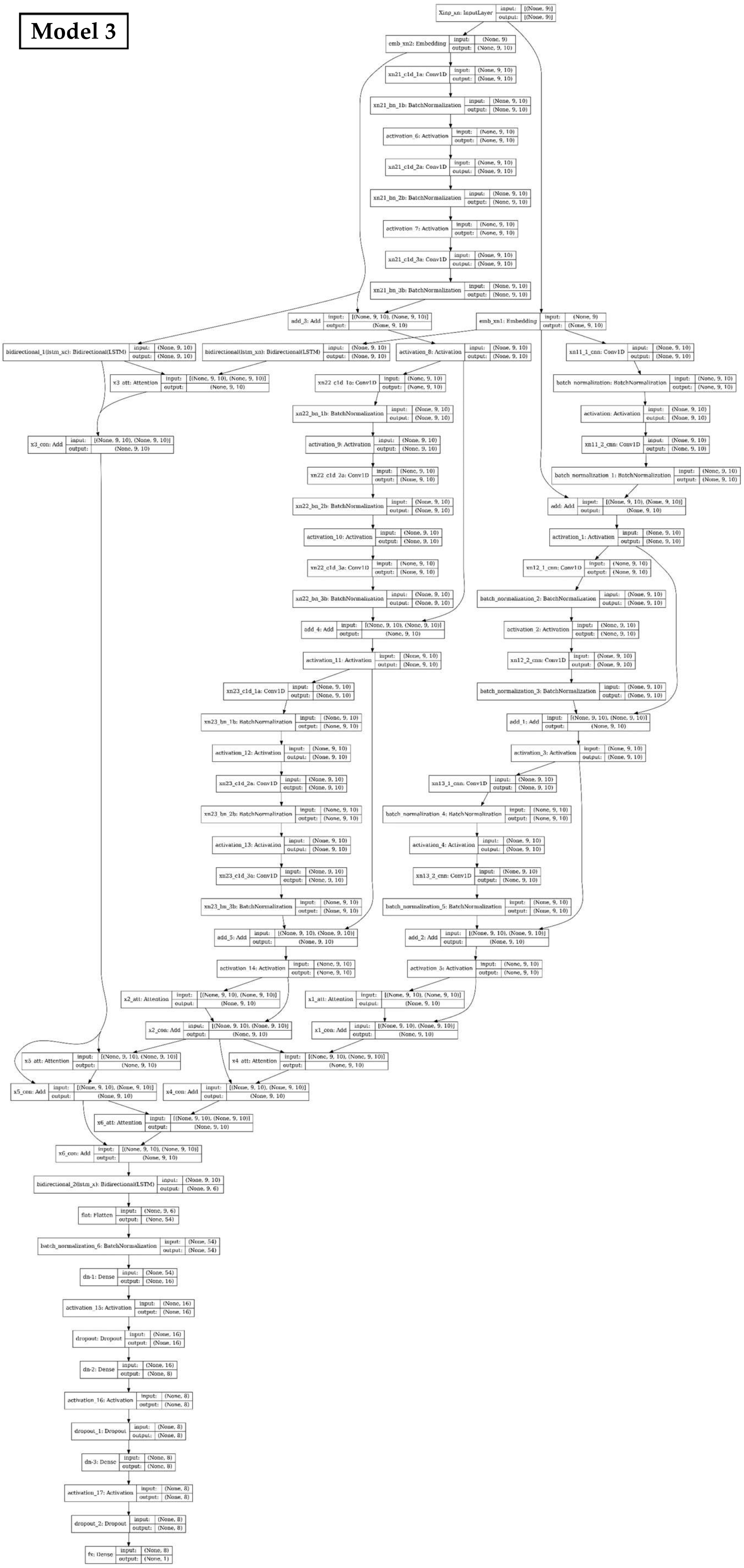
Xinp_xn: InputLayer
emb_xn2: Embedding
xn21_c1d_1a: Conv1D
xn21_bn_1b: BatchNormalization
activation_6: Activation
xn21_c1d_2a: Conv1D
xn21_bn_2b: BatchNormalization
activation_7: Activation
xn21_c1d_3a: Conv1D
xn21_bn_3b: BatchNormalization
add_3: Add
emb_xn1: Embedding
bidirectional_1(lstm_xc): Bidirectional(LSTM)
bidirectional(lstm_xn): Bidirectional(LSTM)
activation_8: Activation
xn11_1_cnn: Conv1D
x3_att: Attention
xn22_c1d_1a: Conv1D
batch_normalization: BatchNormalization
xn22_bn_1b: BatchNormalization
activation: Activation
x3_con: Add
activation_9: Activation
xn11_2_cnn: Conv1D
xn22_c1d_2a: Conv1D
batch_normalization_1: BatchNormalization
xn22_bn_2b: BatchNormalization
add: Add
activation_10: Activation
activation_1: Activation
xn22_c1d_3a: Conv1D
xn12_1_cnn: Conv1D
xn22_bn_3b: BatchNormalization
batch_normalization_2: BatchNormalization
add_4: Add
activation_2: Activation
activation_11: Activation
xn12_2_cnn: Conv1D
xn23_c1d_1a: Conv1D
batch_normalization_3: BatchNormalization
xn23_bn_1b: BatchNormalization
add_1: Add
activation_12: Activation
activation_3: Activation
xn23_c1d_2a: Conv1D
xn13_1_cnn: Conv1D
xn23_bn_2b: BatchNormalization
batch_normalization_4: BatchNormalization
activation_13: Activation
activation_4: Activation
xn23_c1d_3a: Conv1D
xn13_2_cnn: Conv1D
xn23_bn_3b: BatchNormalization
batch_normalization_5: BatchNormalization
add_5: Add
add_2: Add
activation_14: Activation
activation_5: Activation
x2_att: Attention
x1_att: Attention
x2_con: Add
x1_con: Add
x5_att: Attention
x4_att: Attention
x5_con: Add
x4_con: Add
x6_att: Attention
x6_con: Add
bidirectional_2(lstm_x): Bidirectional(LSTM)
flat: Flatten
batch_normalization_6: BatchNormalization
dn-1: Dense
activation_15: Activation
dropout: Dropout
dn-2: Dense
activation_16: Activation
dropout_1: Dropout
dn-3: Dense
activation_17: Activation
dropout_2: Dropout
fx: Dense

Model 4

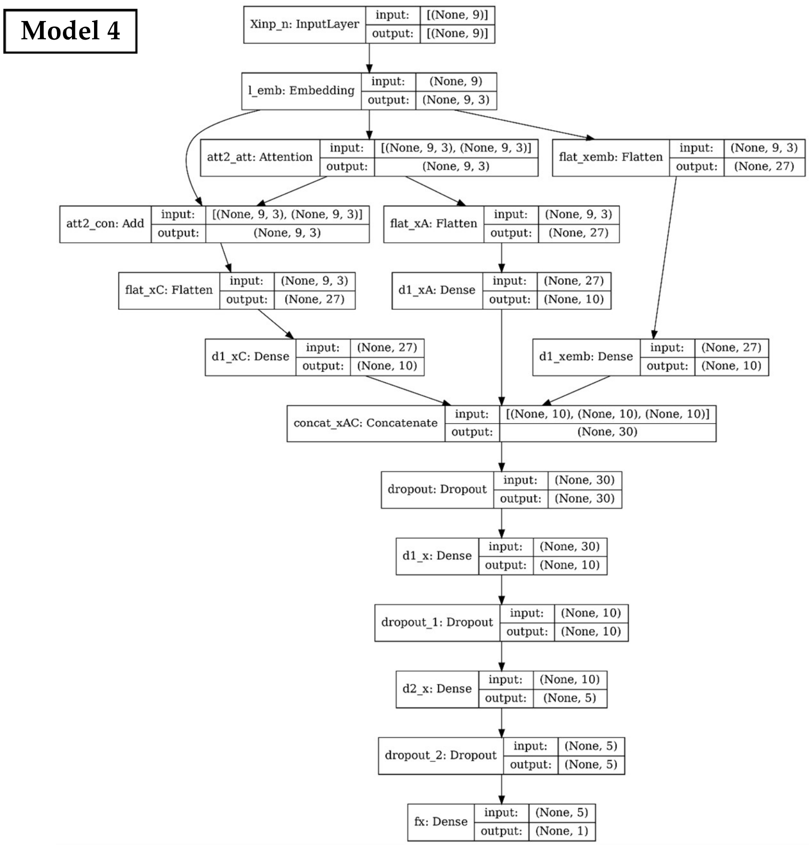

Model 5

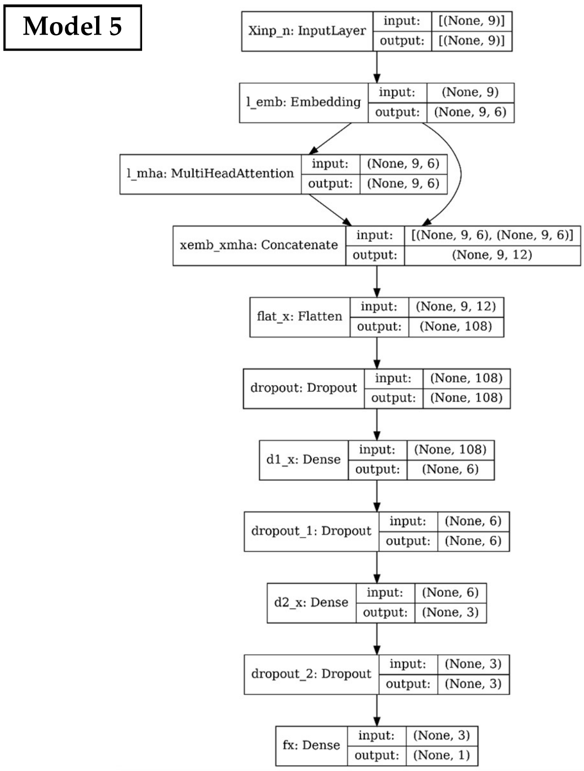

Figure S1. Model architecture of the 5 models (Model 1 to Model 5) in AiCPP. Each box represents one layer. There are embedding layers, CNN layers, LSTM layers, attention layers, etc.

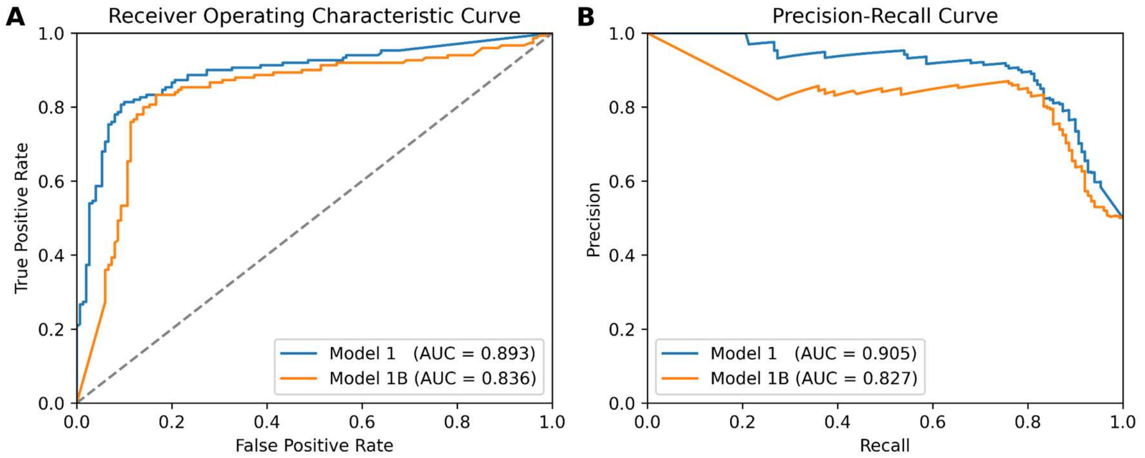

Figure S2. Comparison of the model performance by the ROC-metric with (Model 1) and without (Model 1B) the use of a large number of the 11,046,343 9-mers negative datasets derived from human reference proteins.

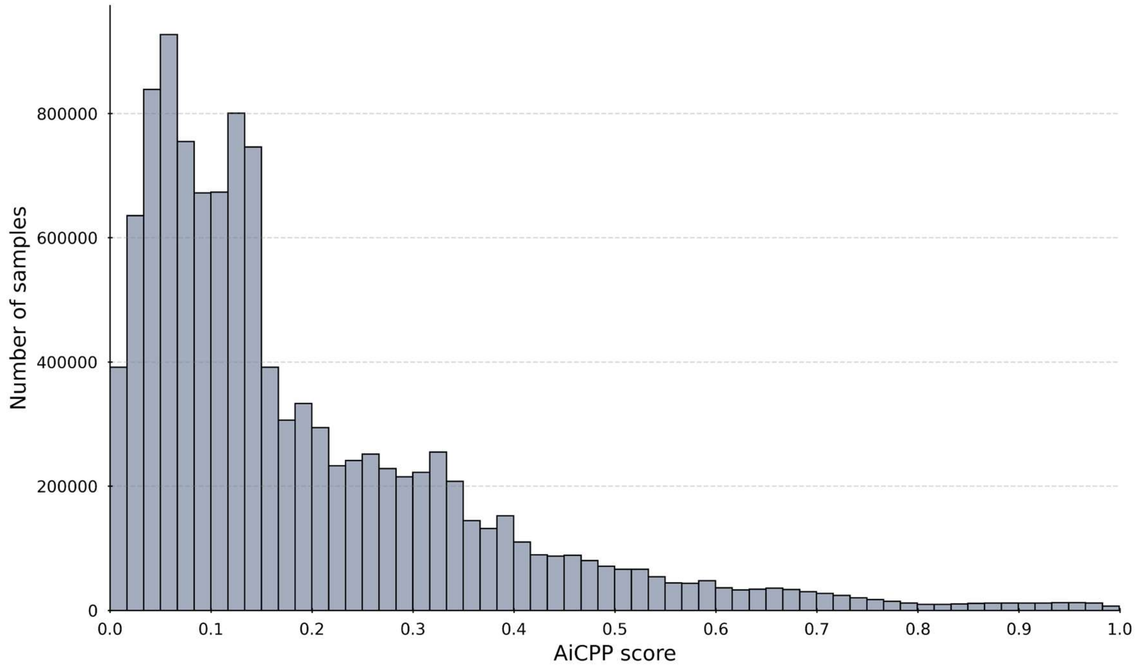

Figure S3. The AiCPP scores for the 11,046,343 9-mers generated from human reference proteins. There are about 770,435 or 6.97% 9-mers with AiCPP scores >= 0.5.

Table S1. Comparison of the metrics of the model performance with (Model 1) and without (Model 1B) the use of about 11 million of a large number of negative datasets derived from human reference proteins.

Models	AUC	MCC	ACC	SEN	SPE	PRE
**Model 1**	**0.893**	**0.711**	**0.853**	0.800	**0.907**	**0.896**
**Model 1B**	0.836	0.640	0.820	**0.833**	0.807	0.812
